# Severe knowlesi malaria with acute neurocognitive impairment: A case report

**DOI:** 10.1016/j.ijregi.2024.100513

**Published:** 2024-12-06

**Authors:** Carta Agrawanto Gunawan

**Affiliations:** Division of Infectious Disease and Tropical Medicine, Department of Internal Medicine, Abdoel Wahab Sjahranie General Hospital/Mulawarman University Faculty of Medicine, Samarinda, Indonesia

**Keywords:** Severe knowlesi malaria, High parasitemia level, Acute neurocognitive impairments

## Abstract

*Plasmodium knowlesi* infection in Indonesia has been reported from Sumatera and Kalimantan Island. *P. knowlesi* has the shortest asexual replication cycle of all *Plasmodium* species, leading to rapidly increased parasitemia levels; however, it usually does not cause high parasitemia. It was reported a severe knowlesi malaria case in a 41-year-old male patient in Samarinda, East Kalimantan Province, Indonesia. The patient was admitted to the hospital with fever and decrease in level of consciousness. Malarial blood smear showed trophozoites and ring forms of *P. knowlesi,* with parasite count 52,000/µl blood, and confirmed by polymerase chain reaction test. The patient was diagnosed as having severe knowlesi malaria with a high parasitemia level, accompanied by acute neurocognitive impairments, including moderate weakness, disorientation, and partial loss of memory that lasted for approximately 3 weeks.

## Highlights

• Knowlesi malaria is only found in Southeast Asia, including Indonesia.
• *Plasmodium knowlesi* has a lower median parasitemia level to cause severe malaria.
• Impaired consciousness is uncommon, and unrousable coma has not been reported.
• Parasite count is important to guide therapy and referral.

## Introduction

*Plasmodium knowlesi* is known as the fifth *Plasmodium* species that can cause malaria in human beings [1,2]. In the period of 2008-2015, there were 418 cases of *P. knowlesi* infection reported from Indonesia [1].

We have reported seven uncomplicated knowlesi malaria cases in East Kalimantan Province in the period of 2018-2021 [3]. We would like to report a severe knowlesi malaria case with a high parasitemia level and impaired consciousness accompanied by acute neurocognitive deficits in Samarinda, East Kalimantan Province, Indonesia in 2023. Recently, to the best of our knowledge, there has not been a published severe knowlesi malaria case from East Kalimantan. In addition, uncomplicated and severe knowlesi malaria is likely under-recognized and under-reported in Indonesia.

## Case report

A male patient, aged 41 years old, was admitted to hospital in Samarinda in September 2023, with intermittent fever for 2 weeks and could not communicate 4 days before admission. On admission, patient had a Glassgow comma scale (GCS) (eye response: 4, verbal response: 2, motor response: 5) score of 11, moderately ill condition, body weight of 55 kg, blood pressure of 110/70 mm Hg, pulse rate of 96 beats per minute, respiratory rate of 22 cycles per minute, and body temperature of 37.7°C.

**Figure 1:**
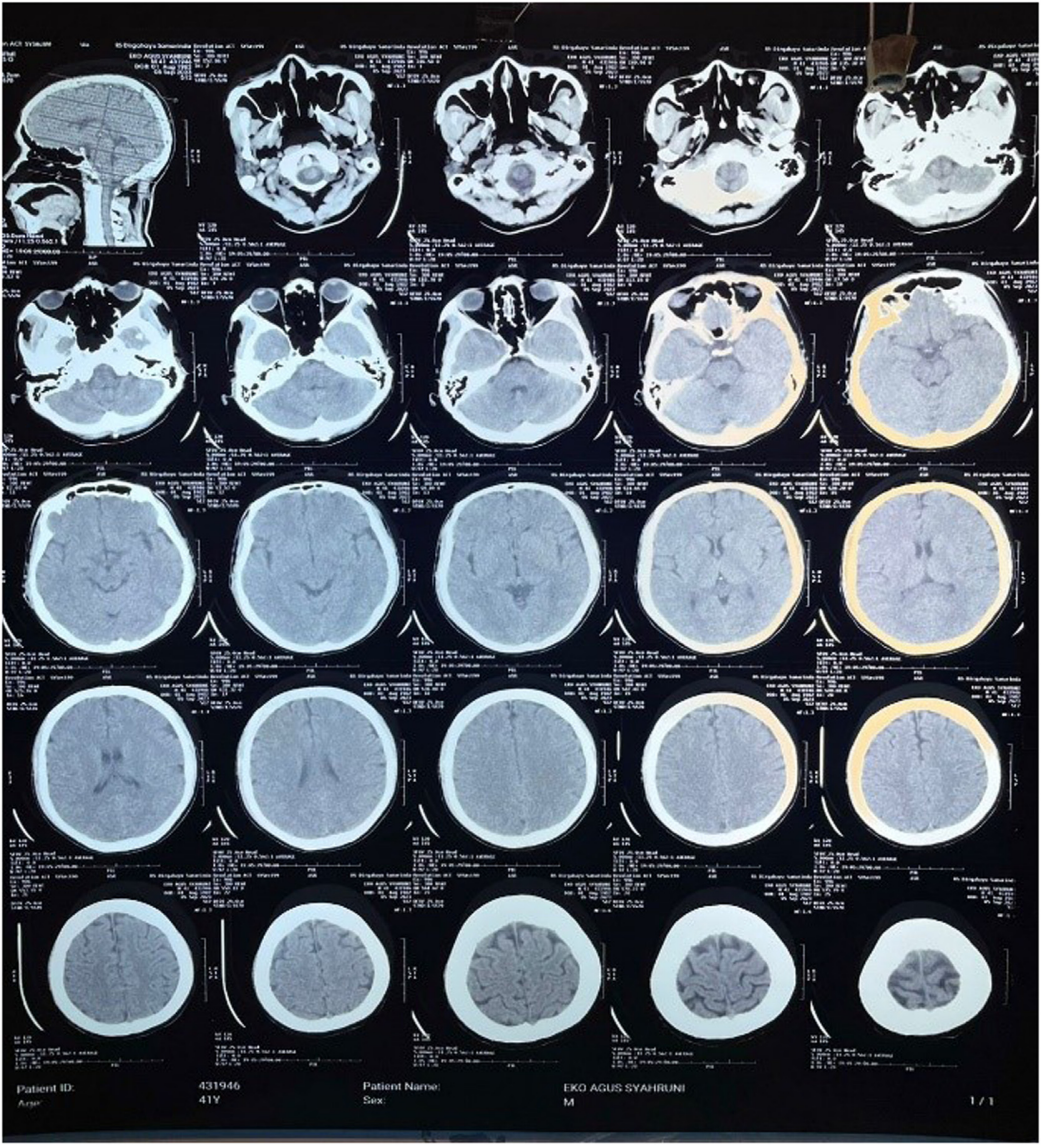
Head computed tomography did not show any focal lesions.

Laboratory examination on admission were as follows: hemoglobin 5.3 g/dl, creatinine 0.75 mg/dl, SGOT 42 IU/l, sodium 143 mmol/l, and potassium 4.7 mmol/l. Peripheral blood smear showed trophozoites and ring forms of *P. knowlesi* (similar to *P. malariae*), with parasite count 52,000/µl blood. Polymerase chain reaction test later showed it was confirmed as *P. knowlesi*. Head computed tomography did not show any focal lesions (Figure 1).

The patient received artesunate injections of 120 mg every 12 hours until three doses, continued with dihydroartemisinin 40 mg/piperaquine 320 mg three tablets per day for 3 days. After three doses of artesunate, the patient's GCS improved to 14. Malarial blood smear 3 days after starting artesunate injection was negative for plasmodium. The patient also received transfusion of four units of packed red blood cells, and the hemoglobin level increased to 11.8 g/dl.

The patient was discharged after 10 days at hospital, with disorientation, partial loss of memory, and moderate weakness. A total of 3 days after discharge, the patient came to outpatient clinic with good improvement in motor function, fully conscious, and with good recovery of memory. The patient came for follow-up 2 weeks later, with satisfying improvement and no neurologic deficits.

## Discussion

In Indonesia, Sumatera and Kalimantan (Borneo) Island are the regions where *P. knowlesi* infections have been reported. The first confirmed *P. knowlesi* case in East Kalimantan Province was reported in 2018.

Besides fever, in knowlesi malaria, headache, muscle pain, nausea, and abdominal pain are frequently found [2]. Severe knowlesi malaria occurred in 6-9% of adult patients [4]. Some frequent complications reported were acute kidney injury, jaundice, acute respiratory distress syndrome, and hypotension [4,5]. *P. knowlesi* has the shortest asexual replication cycle of all *Plasmodium* species, leading to rapidly increased parasitemia levels; however, it usually does not cause high parasitemia [4]. *P. knowlesi* has a lower threshold of parasitemia (>100,000 parasites/µl blood) to be classified as severe malaria with hyperparasitemia [6]. The patient we reported had a high parasite count of 52,000/µl blood but did not fulfill the World Health Organization (WHO) criteria for severe knowlesi malaria with hyperparasitemia. A large prospective study from Sabah, Malaysia showed that severe knowlesi malaria occurred in 53% of patients with parasite count >20,000/ml and 83% in patients with parasite count >100,000/ml [4]. Several prospective studies showed that *P. knowlesi* caused severe malaria with a lower median parasitemia level than severe malaria from *Plasmodium falciparum* [4,7].

In *P. knowlesi* infection, marked reduction in deformability of infected and uninfected red blood cells, endothelial activation, endothelial dysfunction, and glycocalyx degradation are associated with disease severity and are likely contributing to impaired microvascular perfusion and organ dysfunction [7].

This patient had impaired consciousness (GCS 11) but did not meet the WHO unrousable coma definition to define cerebral malaria [6]. Acute altered mental state is uncommonly seen in complicated knowlesi malaria, particularly, in older adults, but deep (arousable) coma (GCS <11) has not been reported [7,8]. This patient also had acute severe anemia with hemoglobin 5.3 g/dl that met the WHO criteria for severe malarial anemia in adult patients (hemoglobin <7 g/dl) that might have contributed to altered mental state.

Artesunate injection is used in the management of severe malaria, including severe knowlesi malaria, with dosage of 2.4 mg/kg body weight intravenous slowly (>2 minutes) for at least three doses [6,9]. If the patient's condition improves after three doses of artesunate, treatment can be continued with oral anti-malaria for 3 days. In this case report, this patient was treated with intravenous artesunate for three doses and continued with 3 days of dihydroartemisinin 40 mg/piperaquine 320 mg. This regimen showed a satisfying outcome for this patient. Parasite count obtained from microscopic examination is important to guide therapy with intravenous artesunate and referral to the highest level of care for severe malaria. Parasitemia >15,000/µl is the best predictor for severe knowlesi malaria (in this case, 52,000/µl), and, in clinical practice, intravenous artesunate is recommended by WHO for all patients with knowlesi malaria with parasite count >15,000/µl [7,8].

## Conclusion

*P. knowlesi* can cause severe malaria with a lower parasitemia level compared with that of *P. falciparum.* Impaired consciousness can occur in knowlesi malaria, particularly, in adult patients, although it is uncommon. To the best of our knowledge, this might be the first severe knowlesi malaria case with acute neurocognitive impairment reported from East Kalimantan Province, Indonesia.

## Declarations of competing interest

The authors declare that they have no known competing financial interests or personal relationships that could have appeared to influence the work reported in this paper.
